# Potential Beneficial Effects of Vitamin D in Coronary Artery Disease

**DOI:** 10.3390/nu12010099

**Published:** 2019-12-30

**Authors:** Christian Legarth, Daniela Grimm, Marcus Krüger, Manfred Infanger, Markus Wehland

**Affiliations:** 1 Department of Biomedicine, Aarhus University, Høegh-Guldbergsgade 10, 8000 Aarhus C, Denmark; chr_brod@hotmail.com; 2 Clinic for Plastic, Aesthetic and Hand Surgery, Otto von Guericke University, Leipziger Str. 44, 39120 Magdeburg, Germany; marcus.krueger@med.ovgu.de (M.K.); manfred.infanger@med.ovgu.de (M.I.)

**Keywords:** vitamin D, cholecalciferol, calcitriol, ischemic heart disease, coronary artery disease

## Abstract

Vitamin D plays a pivotal role in bone homeostasis and calcium metabolism. However, recent research has indicated additional beneficial effects of vitamin D on the cardiovascular system. This review aims to elucidate if vitamin D can be used as an add-on treatment in coronary artery disease (CAD). Large-scale epidemiological studies have found a significant inverse association between serum 25(OH)-vitamin D levels and the prevalence of essential hypertension. Likewise, epidemiological data have suggested plasma levels of vitamin D to be inversely correlated to cardiac injury after acute myocardial infarction (MI). Remarkably, in vitro trials have showed that vitamin D can actively suppress the intracellular NF-κB pathway to decrease CAD progression. This is suggested as a mechanistic link to explain how vitamin D may decrease vascular inflammation and atherosclerosis. A review of randomized controlled trials with vitamin D supplementation showed ambiguous results. This may partly be explained by heterogeneous study groups. It is suggested that subgroups of diabetic patients may benefit more from vitamin D supplementation. Moreover, some studies have indicated that calcitriol rather than cholecalciferol exerts more potent beneficial effects on atherosclerosis and CAD. Therefore, further studies are required to clarify these assumptions.

## 1. Introduction

Cardiovascular disease (CVD) is a major concern of global health. According to the World Health Organization (WHO), CVD is the most common cause of mortality worldwide. Approximately 17.9 million people died from CVD in 2015, with 7.3 million of these deaths due to coronary artery disease (CAD) [1]. Although CAD is formerly considered a disease mediated by lipid accumulation, its pathophysiology is complex, and the exact underlying mechanisms are still unknown. More recent investigations have suggested an additional excessive inflammatory response in the subintimal arterial space followed by thrombus formation [2,3]. Furthermore, several studies have found that blood microparticle levels are elevated in individuals with CAD [4,5]. Different molecules on the surface of the microparticles mediate procoagulant properties that may lead to an acute coronary event [6].

In 2016, the prevalence of CAD in Denmark was estimated to be approximately 160,000 people [7]. Interestingly, new data suggest that vitamin D is a potentially cost-effective treatment agent for CAD [8]. This review will focus on relevant studies in order to investigate whether vitamin D supplementation may exert beneficial effects on atherosclerosis and CAD.

## 2. Literature Search and Investigation

Studies included in this review met the following criteria: participants were adults (>18 years); measured endpoints included risk of myocardial infarction (MI), mortality, plaque burden, CAD events, pulse wave velocity (PWV), adhesion molecules, blood lipids, high-sensitive C-reactive protein (hsCRP) and/or SYNTAX score. Only data from randomized clinical trial (RCT) studies in the publication period from 1 January 2010 to 12 October 2019 were assessed. Only English-language studies that were completed with available results were included. Studies with only stroke or heart failure (HF) as the CVD endpoints were excluded. Moreover, studies with cohorts consisting only of chronic kidney disease (CKD) patients were excluded. The intervention arm was administered calcitriol, cholecalciferol or ergocalciferol.

**Figure 1:**
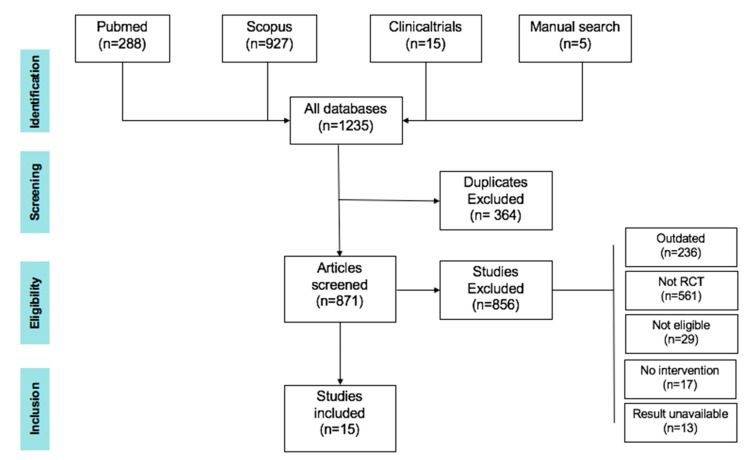
Flowchart of study selection for this review.

To prepare the literature review, the following databases were used: PubMed (https://www.ncbi.nlm.nih.gov/pubmed/), Clinicaltrials.gov (https://clinicaltrials.gov/) and Scopus (https://www.scopus.com). Search items were (‘Coronary Artery Disease’[Mesh] OR ‘Myocardial Infarction’[Mesh]) AND (‘Vitamin D’[Mesh] OR ‘Cholecalciferol’[Mesh]) AND (Clinical Trial[ptyp]), or (‘Vitamin D’[Mesh] OR ‘Cholecalciferol’[Mesh] AND ‘Heart Failure’[Mesh]) AND (‘Myocardial Infarction’[Mesh]), which were applied to the databases to identify studies. A search for ‘Vitamin D’ yielded 81,299 items, ‘Coronary artery disease’ yielded 163,605 items and ‘Vitamin D AND coronary artery disease’ yielded 155 items in PubMed (20 October 2019). There were 927 studies identified in Scopus after performing a search for ‘Coronary artery disease’ OR ‘Myocardial infarction’ AND ‘Vitamin D’ within the time period 1 January 2010 until 12 October 2019. Figure 1 illustrates the flowchart of the study selection.

In order to determine whether vitamin D can reduce vascular inflammation and atherosclerosis by suppression of the NF-κB pathway and be used as a potential treatment agent for CAD in patients with hypovitaminosis D, this review will define CAD and its risk factors, pathophysiology, symptoms and treatment. Next, the physiological role of vitamin D will be described. This review will investigate if vitamin D can be used as a prognostic marker of CAD risk. Finally, the potential role of vitamin D in cardiomyocytes after MI and possible cardioprotective mechanisms will be elucidated.

## 3. Coronary Artery Disease

CAD is an overall group of clinical conditions including stable angina, unstable angina, myocardial infarction (MI) and sudden death [9]. Important complications are heart failure and arrhythmia. The symptoms of CAD are the result of an inadequate blood supply of the heart caused by the obstruction of a coronary artery. MI is the most common manifestation of CAD and is due to the disruption of a vulnerable atherosclerotic plaque or the erosion of the coronary artery endothelium. Upon rupture, the atherosclerotic plaque releases thrombogenic contents, initiating a coagulation cascade. This hypercoagulable state could especially contribute to the rupture of additional vulnerable fibroatheromas leading to more than one culprit lesion. MI finally ends in an irreversible necrosis of myocardial cells that is detectable by an elevation of cardiac biomarkers [10,11].

### 3.1. Pathophysiology of Atherosclerosis

The underlying mechanisms of atherosclerosis can be divided into two parts: formation of a stable plaque and transition into an unstable plaque. The first process involves endothelial erosion with endothelial activation and denudation [12]. This endothelial dysfunction results in the deposit of low-density lipoprotein (LDL) molecules in the vascular intima [13], which leads to the formation of fatty streaks and eventually stable plaques. Lipoxygenases and myeloperoxidases oxidize the LDL molecules in the vessel wall. This oxidation attracts and stimulates activated macrophages [12]. These macrophages may induce apoptosis of endothelial cells and form a thin fibrous plaque cap that separates the lipid core from the lumen. The plaque consists of degraded smooth muscle cells, endothelial cells, foam cells, cellular debris, lymphocytes and modified lipids [14]. This mechanism is referred to as atherosclerosis and causes a gradual narrowing of the lumen. Atherosclerosis is considered to be a chronic inflammatory process in the vessel wall [15].

Subsequently, atherosclerosis may be followed by rupture of the vulnerable plaque cap. This exposes the lipid core to the vessel lumen. The atheromatous mass is now thrombogenic and causes platelet activation and finally coronary occlusion [16]. Coronary artery narrowing or occlusion may cause the symptoms of angina due to the onset of ischemia.

### 3.2. CAD Symptoms

The CAD symptom spectrum of angina can manifest itself in multiple ways. According to the European Society of Cardiology (ESC) [17], the typical symptoms include discomfort, pain, nausea, fatigue, restlessness, burning, shortness of breath and uncomfortable chest pressure. The sensation of discomfort is most often located at the chest or near the sternum. However, this pain may also be localized between shoulder blades, at the jaw, teeth, in either arm or at the wrist and fingers. In most cases, the pain has a duration of ≤ 10 min and is triggered by physical exercise. Table 1 shows the typical characteristics of pain due to CAD.

### 3.3. CAD Prognosis

A serious complication to acute MI is the progression of heart failure (HF) with reduced function of the left ventricle (LV) [18]. The estimated risk of LV systolic dysfunction after MI is about 40% [19]. Pathogenesis is partly based upon excessive β-adrenergic activation post-MI. This mechanism is complex and promotes cardiomyocyte growth, vasoconstriction and cardiac injury. Subsequently, this might lead to cardiac remodeling and LV dysfunction [20].

### 3.4. Diagnosis of CAD

A thorough prior diagnosis is crucial. This section gives an overview of basic test procedures in patients with suspected diagnosis of CAD. Table 2 depicts some of the useful procedures in diagnosis of CAD.

### 3.5. Current Treatment Options of CAD

Treatment of CAD must focus on both acute treatment and secondary prophylaxis. Smoking, physical inactivity, high body mass index (BMI), diabetes mellitus, hypertension, excessive dietary fat and genetic dispositions are some of the known risk factors associated with CAD [26]. They all contribute to the lifelong atherosclerotic process and increase the risk of ischemic events. Thus, public health approaches with a focus on smoking cessation, healthy diet, stress reduction, physical exercise and antihypertensive treatment are of great importance [16]. In addition, Ornish et al. [27] showed a 7.9% relative reduction of coronary artery stenosis after five years with the intensive lifestyle interventions mentioned above.

The pharmaceutical treatment of CAD aims to reduce CVD and improve survival. More drug classes can be included in the treatment regimen. Table 3 illustrates an overview of drugs used in the treatment of CAD.

Favaloro et al. (1971) [39] were the first to describe surgical techniques with vein grafts in acute MI patients to re-establish blood supply to the myocardium. Since then, great progress has been made in order to improve survival in acute coronary syndrome (ACS) patients.

- Percutaneous coronary intervention (PCI): The PCI procedure is an effective strategy for revascularization in CAD patients with both acute and stable forms. The intervention is performed by inserting a guidewire catheter into the femoral or radial artery. The guidewire is guided to the coronary artery, where the thrombosis is located. Here, a balloon is inflated, and, for example, a metallic stent might be inserted in order to prevent reinfarction. Stents can either be bare metal or drug-eluting (everolimus, zotarolimus, etc.) to minimize restenosis [17,40]. In principle, PCI is the preferred procedure in patients with ST-segment elevation myocardial infarction (STEMI) within 12 h of symptom onset [41]. In addition, patients with non-ST-segment elevation myocardial infarction (NSTEMI) might be offered PCI within 48 h of symptom onset if no relevant comorbidity is present.
- Coronary artery bypass grafting (CABG): The CAGB procedure is considered more invasive compared to PCI. The procedure includes bypassing stenosed coronary arteries [42]. Thus, vein or artery grafts are used to anastomose occluded vessels. According to the SYNTAX study, the CABG strategy is preferable in more complex multivessel occlusions [43].

## 4. Vitamin D

Vitamin D is mainly synthesized endogenously, when the skin is exposed to ultraviolet radiation from sunlight. Since Askew et al. [44] first isolated vitamin D in 1932, much knowledge has been gained to understand the functions of this vitamin.

### 4.1. Vitamin D Metabolism

**Figure 2:**
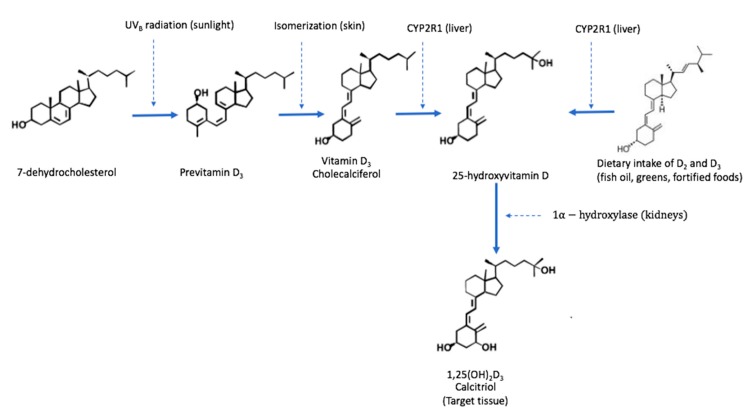
The metabolism of vitamin D in humans; modified from [46].

The active form of vitamin D (named 1,25(OH)_2_D_3_) is based upon a modified steroid scaffold with lipophilic properties [45]. Its chemical structure contains a secosteroid with an open B-ring. Figure 2 shows a schematic overview of vitamin D metabolism.

Typically, the marker 25-hydroxyvitamin D (25(OH)D) is used as a surrogate endpoint of vitamin D status in plasma [47]. Historically it has been difficult to establish evidence-based recommendations for optimal plasma levels of vitamin D. Several health regulatory agencies have published slightly different definitions of vitamin deficiency based on the serum levels of 25-hydroxyvitamin D. An international consensus on the definition of vitamin D deficiency and sufficiency is lacking. Table 4 summarizes the definitions of selected health organizations and the Mayo Clinic [48,49,50,51,52,53].

Vitamin D is important for bone health and calcium homeostasis in humans. However, recent studies have implied that vitamin D has extraskeletal functions as well. Hewison et al. demonstrated that the enzyme 25(OH)-hydroxyvitamin D_3_-1α-hydroxylase is present in various extrarenal tissues [54], elucidating the local synthesis of active 1,25(OH)_2_D_3_. These findings suggest that vitamin D has autocrine and paracrine functions [54]. This probably exerts a positive impact on cardiovascular health and the immune system and prevents the development of diabetes mellitus [55].

### 4.2. Cardiovascular Effects of Vitamin D

In a recent review summarizing the current knowledge of the effects of vitamin D on cardiovascular disease, Saponaro et al. [56] demonstrated that this scientific field has drawn considerable attention in recent years. As detailed below, vitamin D deficiency is associated with hypertension [57], which is a risk factor in the atherosclerotic process. Moreover, in vitro models have been used to understand the possible mechanistic effects of vitamin D in CAD progression [58] and the suppression of renin synthesis [59]. Al-Ishaq et al. [60] have stated that vitamin D deficiency activates the renin-angiotensin-aldosterone system, which might lead to cardiac hypertrophy and increased CVD risk. However, RCTs and Mendelian studies have been inconclusive regarding the causality of vitamin D supplementation and improved cardiovascular outcomes [61].

#### 4.2.1. Vitamin D and Essential Hypertension

In a review on vitamin D and essential hypertension [62], it was pointed out that, based on data from the third National Health and Nutrition Examination Survey (NHANES III), vitamin D deficiency is associated with essential hypertension [63,64]. In addition to these epidemiological findings, Yuan et al. demonstrated that vitamin D can suppress renin synthesis in vitro [59]. Nevertheless, RCTs performed to assess the impact of vitamin D supplementation on hypertension showed equivocal results [62]. This can, in part, be attributed to suboptimal study designs. 

#### 4.2.2. Association between Serum Vitamin D and Myocardial Injury

Using NHANES III data, Ahmad et al. [65] examined a possible association between serum vitamin D concentration and subclinical myocardial injury. In this cross-sectional study, recruited individuals were sought to be representative of the background population [66]. Hence, 8561 participants underwent a 12-lead ECG to visualize the electrical conduction of the heart. Participants with earlier diagnosed CVD were excluded, and thus 6079 participants were included for this analysis in the period between 1988 and 1994. To evaluate subclinical myocardial injury (SC-MI) in ECG measurements, the objective multivariate tool named the Cardiac Infarction Injury Score (CIIS) [67] was chosen. Participants were divided into three tertiles based on their serum levels of 25(OH)D (<20, 20–30 and >30 ng/mL). 

The first group (serum-25(OH)D < 20 ng/mL) showed a prevalence of SC-MI = 23.0%, while the second group (serum-25(OH)D 20–30 ng/mL) had a prevalence of SC-MI = 21.1%. The third group (reference) with serum-25(OH)D > 30 ng/mL had a prevalence of SC-MI = 19.5%. A comparison of groups one and three revealed that SC-MI was inversely associated with 25(OH)D levels with an odds ratio (OR) of 1.27 (95% CI: 1.04–1.55) after adjustments for potential confounders [65]. Hence, the study found a significant incremental increase in SC-MI prevalence associated with vitamin D deficiency (*p* = 0.04). 

Verdoia et al. [68] conducted another cross-sectional study to investigate the relationship between serum 25(OH)D-levels and CAD. The examined cohort comprised 1484 patients, all of whom underwent elective coronary angiography. The results showed that vitamin D deficiency is significantly associated with the severity of CAD. Comparing the odds of CAD in patients with severe hypovitaminosis D (<10 ng/mL) and patients with normal vitamin D status yielded an adjusted OR of 1.73 (95% CI: 1.18–2.52).

The strengths of these studies can be attributed to the large sample size. Moreover, data included in the NHANES III study were derived from a sample group without prior CVD history. So far, the NHANES III survey is the most comprehensive study, where both information on serum 25(OH)D-levels and markers of myocardial injury can be extracted. However, the methodological limitations include potential confounding factors, as the evaluations of exposure and outcomes were not temporally separated. Likewise, seasonal variation in serum 25(OH)D-levels might be a concern. This is due to fact that information is lacking about the time of year at which the participants had blood samples collected [65]. Even though both studies found strong associations between vitamin D status and CAD, this does not necessarily substantiate causality. 

#### 4.2.3. Impact of Vitamin D on Cardiac Function after MI

Le et al. [69] conducted an in vivo study to explore the effects of vitamin D on cardiac function in post-MI mice. One group of 1,25(OH)_2_D_3_ supplemented mice (*n* = 5) was compared to non-vitamin D supplemented controls (*n* = 5). The experimental mice were offered an optimal diet and husbandry conditions. At the start of the study, MI was induced in all mice by ligating the left anterior descending (LAD) artery. Subsequently, the intervention group was administered calcitriol 0.6 μg/day/kg for 14 days and examined by echocardiography and histological analysis. The results showed a significant reduction in the fibrotic scar area in the LV in the intervention arm compared with controls (*p* < 0.05). Likewise, LV wall thinning after MI was attenuated in calcitriol- supplemented mice versus controls (*p* < 0.05). Le et al. [69] performed an in vitro experiment to provide a mechanistic explanation for these findings (i.e., that vitamin D suppresses cell cycle progression in cCFU progenitor cells). Thus, cardiac myofibroblast differentiation might be decreased after calcitriol supplementation. 

#### 4.2.4. Possible Mechanisms behind Vitamin D Effects on CAD

**Figure 3:**
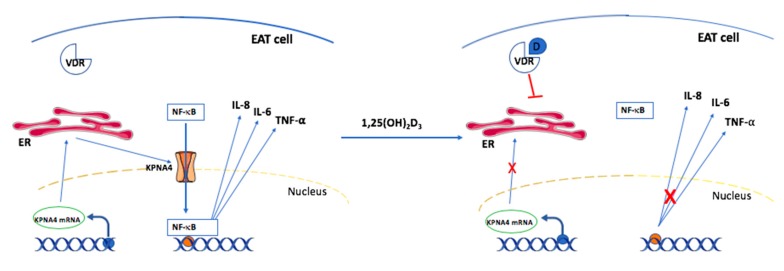
Suppression of NF-κB pathways by vitamin D supplementation (modified from [53]). EAT cell: epicardial adipose tissue cell; VDR: vitamin D receptor; KPNA4: karyopherin α4; mRNA: messenger ribonucleic acid; NF-κB: nuclear factor kappa B; D: 1,25(OH)_2_-vitamin D3; IL-6: interleukin 6; IL-8: interleukin 8; TNF-α: tumor necrosis factor-α; ER: endoplasmic reticulum.

To clarify possible underlying mechanisms of vitamin D effects on CAD, Chen et al. [58] performed a study in swine. Here, epicardial adipose tissue (EAT) cells were extracted and cultured as preadipocytes in vitro. Interestingly, this study indicated that vitamin D suppresses the nuclear factor ‘kappa-light-chain-enhancer’ of the activated B-cells (NF-κB) pathway and thereby attenuates the progression of CAD. Figure 3 depicts how vitamin D interferes with the NF-κB pathway.

EAT cells are deeply involved in the progression of coronary atherogenesis mediated through the synthesis of local inflammatory cytokines [70]. First, KPNA4 mRNA is transcribed in the nucleus and transported to the endoplasmic reticulum in the cytosol, where translation takes place. Subsequently, the nascent KPNA4 protein is released and incorporated in the nuclear membrane. KPNA4 is a membrane transporter that is responsible for shuttling NF-κB from the cytosol to the nucleus [71]. NF-κB acts as a transcription factor in the nucleus via binding to different κB elements [72], which promote the transcription of proinflammatory cytokines such as IL-6, IL-8 and TNF-α. These cytokines are involved in the progression of atherogenesis in coronary arteries [73,74]. 

Interestingly, it appears that liganded 1,25(OH)_2_-vitamin D_3_-VDR actively suppresses the transcription and translation of KPNA4 in EAT cells. The reduced expression of KPNA4 leads to compromised shuttling of NF-κB into the nucleus. Hence, sufficient levels of intracellular 1,25(OH)_2_-vitamin D_3_ are capable of reducing the inflammatory response in the atherosclerotic process. This might to some extent be a mechanistic explanation as to how vitamin D deficiency is linked to CAD. However, it still remains to be elucidated how vitamin D_3_ indirectly or directly suppresses KPNA4 transcription [58].

### 4.3. Vitamin D Supplementation and CAD

A comprehensive review of relevant studies has been prepared in order to investigate whether vitamin D supplementation may exert beneficial effects on atherosclerosis and CAD. All eligible RCTs for this review are listed in Table 5.

## 5. Discussion

Overall, this review attempted to elucidate whether vitamin D supplementation could be beneficial as a treatment agent in CAD patients. A possible mechanistic link was provided by Chen et al. [58], who explained how vitamin D alters the inflammatory response of CAD through suppression of the NF-κB pathway. Likewise, Le et al. [69] suggested that calcitriol might decrease fibroblast differentiation in progenitor cCFU cells after MI. However, this study was conducted in mice, which might make it problematic to directly transfer these findings to the human organism. Overall, these results are consistent with epidemiological studies reporting serum 25(OH)D to be inversely correlated with CAD and myocardial injury [65,68]. However, the possibility of unknown confounding factors in these cross-sectional studies cannot be excluded. Hence, randomized prospective studies are in high demand. 

Table 5 shows recent the RCTs that have attempted to address this issue. Nevertheless, the results are ambiguous. The two large scale studies [75,78] failed to demonstrate a beneficial effect of vitamin D on MI risk and CVD events. These studies must be given greater weight due to the large sample size. Interestingly, Scragg et al. [75] used monthly bolus doses of 100,000 IU in their study. One consideration worth following with this study design is the bioavailability of vitamin D. A high-dose intervention with a long dosage interval might be a suboptimal study design [90]. All RCTs that used this study design failed to demonstrate major benefits of vitamin D supplementation, even though plasma levels were restored [75,79,80,82,86].

Hin et al. [76] found no cardiovascular benefits of daily cholecalciferol therapy. Remarkably, plasma levels of 25(OH)D were above 50 nmol/L at baseline and 12 months after the intervention in both the intervention arm and controls. According to the Danish Health Authority [52], the threshold of vitamin D sufficiency is achieved at plasma concentrations between 50–160 nmol/L. Thus, possible additional cardiovascular benefits might be difficult to detect in this sample group.

The two studies by Seibert et al. [77] and Sokol et al. [84] have more similarities. Both studies had a study period of 12 weeks and did not show significant changes in endothelial markers, BP, inflammation or blood lipids. Perhaps a longer duration of follow-up in these studies would have clarified the effect of this intervention.

Only four small RCTs succeeded in showing major cardiovascular improvements following vitamin D supplementation. Wu et al. [81] examined whether daily supplementation of 0.5 μg calcitriol for six months could improve CAD. The results revealed a significantly decreased SYNTAX score (−3.9; *p* < 0.001) and reduced vascular inflammation. This was the only RCT to employ the administration of calcitriol (1α, 25-(OH)_2_D_3_), which is the active form of vitamin D. This vitamin D analogue is more potent compared to cholecalciferol and might be more suitable treating vitamin D insufficiency [91]. Therefore, it could be speculated as to whether this analogue is more effective in exerting positive effects on atherosclerosis and CAD. Nevertheless, further studies are needed to investigate if calcitriol is a better treatment agent in cardiovascular disease. 

In a sub-study of the Women’s Health Initiative, Manson et al. [87] did not find evidence for reduced coronary calcification after seven years of cholecalciferol treatment. It is important to state that this RCT did not obtain information about vitamin D status in participants.

In a small study by Arnson et al. [85], five days of cholecalciferol treatment attenuated some inflammatory and endothelial markers (CRP, VCAM-1 and IL-6). Likewise, Raygan et al. found reduced vascular inflammation (hsCRP) and metabolic improvements in diabetic patients supplemented with 12 weeks of vitamin D [88,89]. Low calcium intake and low vitamin D status have been associated with obesity and diabetes mellitus [92,93]. This hints towards a tendency for better cardiovascular outcomes with vitamin D supplementation in certain patient subgroups, such as type 2 diabetics. Nevertheless, other studies in diabetic type 2 patients failed to demonstrate any effects on vascular inflammation [80,82,83]. In addition, the role of vitamin D in Ca^2+^-mediated apoptosis in obesity indirectly supports the recommendation to reach an optimal vitamin D status [94].

In general, the results in this field are conflicting. A comprehensive 2019 meta-analysis by Barbarawi et al. [95] including 83,000 participants did not find beneficial cardiovascular outcomes following vitamin D supplementation. However, further studies are needed to clarify if special subgroups could benefit from this intervention.

## 6. Conclusions

CAD is one of the most prevalent cardiovascular diseases. This disease is mainly caused by the progression of atherosclerosis. Predisposing factors represent a complex interaction between lifestyle, environmental and genetic contributors. Recent large-scale observational studies have demonstrated a strong inverse correlation between plasma levels of 25(OH)D and coronary atherosclerosis. Interestingly, in vitro studies suggest that vitamin D may attenuate CAD through the downregulation of the NF-κB pathway. However, the results obtained from a review of relevant RCTs presented here did not clearly show cardiovascular improvements following cholecalciferol supplementation.

Only a few RCTs have supported the hypothesis of the benefits of vitamin D in the treatment of CAD. In one study that employed calcitriol as the intervention, the results indicated a significant reduction in CAD and vascular inflammation. Hence, future studies could focus on the effects of more potent vitamin D analogues, such as calcitriol. Likewise, considerations of sufficient doses are important to conducting optimally designed studies. Finally, future studies may consider if certain subgroups such as type 2 diabetics with vitamin D insufficiency are more suitable for vitamin D supplementation.

## Figures and Tables

**Table 1:** Characteristics of coronary artery disease (CAD) symptoms, modified from [17].

Classification	Characteristics
Typical angina	Constricting sensation in front of chest or shoulder, neck, jaw or arm. Symptoms relieved by nitrates or rest ≤ 5 min. Triggered by physical exertion.
Atypical angina	Meets only two of the characteristics above.
Non-anginal chest pain	Meets none or just one of the characteristics above.

CAD may result in stable angina, unstable angina, ST-elevation myocardial infarction (MI) or non ST-elevation myocardial infarction.

**Table 2:** Diagnostic tools for the diagnosis of CAD.

Procedure	Explanation
Electrocardiogram (ECG) [21]	ECG plays a key role in the initial diagnosis in patients presenting with angina symptoms. A resting 12-lead ECG may reveal abnormalities that support the diagnosis of MI or myocardial ischemia. ST-segment deviations (depression/elevation) may visualize myocardial injury.
Biochemical tests [17,22]	This procedure may include blood samples with a lipid profile, fasting glucose and glycated hemoglobin (HbA1c), full blood count and plasma creatinine. Furthermore, it is essential to measure myocardial injury markers such as troponin I, troponin T and creatine kinase myocardial band (CK-MB).
Echocardiography [23]	An echocardiography might be performed as a stress test under physical exercise or under concomitant administration of medication such as dipyridamole or dobutamine. This procedure might reveal areas in the left ventricle (LV) with wall abnormalities or hypocontractility.
Cardiovascular magnetic resonance (CMR) [24]	This procedure utilizes electromagnetic waves for the imaging of heart and coronary vessels. This technique can be used to assess myocardial viability after MI [25].
Coronary catheterization and angiography [11]	As recommended by the 2019 European Society of Cardiology (ESC) guidelines, this technique is now used in cases of inconclusive non-invasive tests and for patients with a high clinical likelihood and severe symptoms refractory to medical therapy or high event risk. A catheter is guided from a peripheral artery to the coronary artery. Subsequently, a contrast medium is injected, and coronary arteries are visualized by X-ray.

**Table 3:** Overview of medications used for CAD.

Class	Indications	Mechanism	Drugs
Beta-adrenoceptor antagonists [28]	arrhythmia, hypertension, post-MI, angina pectoris, CAD	Act through the blockade of beta-adrenoceptors in cardiac muscle cells and vascular SMCs. Lead to decreased heart rate and cardiac output (CO). Secondarily, antagonism of β1-adrenoceptors will cause relaxation of vascular smooth muscle cells, induce vasodilation and lower total periphery resistance (TPR).	Metoprolol Propranolol Carvedilol Atenolol Nebivolol
Acetylsalicylic acid (ASA) [29]	acute MI, CAD, prevent re-thrombosis	Irreversible inactivation of cyclooxygenase (COX-1, -2) enzymes. This inhibition promotes a blockade of thromboxane synthesis, which decreases platelet activation.	Aspirin
Statins [30,31]	hyperlipidemia, dyslipidemia, post-MI	Inhibition of 3-hydroxy-3-methyl-glutaryl-coenzyme A reductase enzyme (HMG-CoA reductase), which catalyzes the transformation of HMG to mevalonic acid, thereby reducing intracellular cholesterol concentrations, which leads to the upregulation of LDL surface receptors. Eventually, plasma concentrations of LDL and total cholesterol fall with statin treatment.	Simvastatin Rosuvastatin Atorvastatin Pravastatin
Nitrates [32,33]	angina pectoris, CAD	Promote the release of nitric oxide (NO) in smooth muscle cells in blood vessels. High intracellular NO concentrations activate guanylyl cyclase (GC). Eventually, this results in decreased intracellular calcium concentrations. This causes dilatation of the coronary arteries, afterload reduction and increased angina threshold.	Glyceryl nitrate Isosorbide dinitrate Isosorbide mononitrate
Angiotensin-converting enzyme inhibitors (ACE-inhibitors) [34,35]	hypertension, LV heart failure (HF), heart failure post- MI, angina pectoris	The enzyme ACE hydrolyzes angiotensin I to the active form angiotensin II (Ang II). Effects carried out by Ang II are decreased by the inhibition of ACE. This promotes vasoconstriction, upregulation of aldosterone secretion in the kidneys and fibrosis in cardiac cells, resulting in vasodilation, reduced TPR and reduced preload and afterload.	Enalapril Captopril Ramipril Trandolapril
Calcium channel blockers [36]	hypertension, angina pectoris	Calcium channel blockers act through the blockade of L-type Ca^2+^ channels in vascular smooth muscle cells. The inhibition of voltage-gated calcium channels results in the decreased release of Ca^2+^ into the cytoplasm. This causes vasodilatation and a fall in TPR and blood pressure. Moreover, non-dihydropyridine derivatives (such as diltiazem and verapamil) have a direct effect on cardiac muscle cells. This leads to subsequent negative inotropy and negative chronotropy.	Diltiazem Verapamil Felodipin Amlodipin Lercadipin
Anti-anginal [37]	chronic angina pectoris	Acts through the inhibition of late influx sodium channels (INa) in cardiomyocytes. Thus, calcium overload is attenuated by reduced sodium/calcium exchange in cardiac myocytes. Subsequently, the oxygen demand is reduced and cardiac output improves.	Ranolazine
Anti-platelet medications [38]	MI, stroke, UAP, stent patients	The mechanism of action is carried out via the blockade of the Gi-protein coupled P2Y12 receptor. This causes inhibition of the intracellular PI3K pathway and diminished platelet activation and aggregation.	Clopidogrel Ticagrelor Prasugrel

**Table 4:** Definition of Vitamin D status (25(OH)D concentration) by different health authorities.

Health organization	Optimal	Insufficiency	Deficiency	Reference
Endocrine Society	≥30 ng/mL (75 nmol/L), preferred range: 40–60 ng/mL	21–29 ng/mL	≤20 ng/mL (50 nmol/L)	[48]
The Institute of Medicine (Health and Medicine Division of the National Academies)	≥20 ng/mL	12–20 ng/mL	<12 ng/mL	[49]
The American Association of Clinical Endocrinologists	30–50 ng/mL	20–29 ng/mL	<20 ng/mL	[50]
Mayo Clinic	>20 ng/mL	11–20 ng/mL	≤10 ng/mL	[51]
Danish Health Authority	20–64 ng/mL (50–160 nmol/L)	10–20 ng/mL (25–50 nmol/L)	<10 ng/mL (25 nmol/L)	[52]
ECTS working group	≥20 ng/mL (50 nmol/L)	N/A	<20 ng/mL (50 nmol/L) Severe: <12 ng/mL (30 nmol/L)	[53]

25(OH)D concentration is given in ng/mL (1 nmol/L = 0.4 ng/mL).

**Table 5:** Reviewed studies examining the effects of vitamin D supplementation on CAD and atherosclerosis.

Title, Study Identifier	Design	Endpoints	Objective	Conclusions
Effect of monthly high-dose vitamin D supplementation on cardiovascular disease in the vitamin D assessment study: a randomized clinical trial. (ViDA) [75] ACTRN12611000402943.	Randomized Double-blinded Placebo-controlled *n* = 5110 Median follow-up = 3.3 years.	Cardiovascular disease (CVD) events Mortality MI	To investigate if monthly supplementation of cholecalciferol (100,000 IU) could prevent CVD events in vitamin D insufficient patients.	Intervention with vitamin D supplementation significantly raises mean serum 25(OH)D-levels, when compared to placebo. However, this study found no beneficial effects of cholecalciferol supplementation on CVD risk or mortality.
Optimum dose of vitamin D for disease prevention in older people: BEST-D trial of vitamin D in primary care. [76] EudraCT number: 2011-005763-24a	Randomized Placebo-controlled Parallel group assignment Double-blinded *n* = 305	BP PWV Cholesterol Heart rate	To evaluate the effects of daily supplementation for one year of cholecalciferol (4000 IU or 2000 IU) on disease risk and biochemical markers in healthy elderly people.	Vitamin D supplementation restores serum levels 25(OH)D, but no significant changes in CVD risk factors, arterial stiffness, blood lipids or blood pressure were observed after the intervention.
Vitamin D3 supplementation does not modify cardiovascular risk profile of adults with inadequate vitamin D status. [77] NCT01711905	Randomized Placebo-controlled Double-blinded *n* = 106	BP Blood lipids Heart rate Renin	To investigate if daily supplementation for 12 weeks of cholecalciferol (800 IU/day) could decrease blood pressure, heart rate and other cardiovascular risk markers in healthy participants.	Vitamin D did not improve cardiovascular risk markers in this study, even though serum 25(OH)D levels were restored.
Vitamin D supplements and prevention of cancer and cardiovascular disease. (ViTAL) [78] NCT01169259	Randomized Placebo-controlled Double-blinded *n* = 25,871	Mortality CVD events MI	To elucidate if daily supplementation for an average of 5.3 years of cholecalciferol (2000 IU/day) could reduce the risk of major cardiovascular events and invasive cancer in a normal population.	Vitamin D supplementation did not improve cardiac health, even though serum 25(OH)D levels were restored. Furthermore, no reduction in mortality was observed. The risk of MI was not significantly reduced in the intervention group (hazard ratio = 0.96 (95% CI: 0.78–1.19).
Effect of vitamin D in the prevention of myocardial injury following elective percutaneous coronary intervention (PCI): a pilot randomized clinical trial. [79] IRCT201402078307N6	Randomized Placebo-controlled Double-blinded *n* = 99	Cardiac injury CK-MB cTnl	To investigate if a 300,000 IU dose of cholecalciferol given before PCI could prevent myocardial injury.	No significant changes in cardiac injury markers were noted. However, the biomarkers CK-MB and cTnI were non-significantly improved (*p* > 0.05). The cardiac parameter hsCRP was statistically altered in favor of vitamin D when the two groups were compared at baseline and 24 h later. (−2.1 and −3: *p* = 0.045). No incidence of major adverse cardiovascular events (MACE) occurred in the study groups, thereby effects on incident CAD events could not be evaluated.
Effects of vitamin D2 or D3 supplementation on glycemic control and cardiometabolic risk among people at risk of type 2 diabetes: results of a randomized double-blind placebo-controlled trial. [80] EudraCT 2009-011264-11	Randomized Placebo-controlled Double-blinded *n* = 340	HbA1c PWV hsCRP Blood lipids	To investigate whether monthly supplementation of either ergocalciferol (100,000 IU/month) or cholecalciferol (100,000 IU/month) orally administered for four months could improve cardiometabolic parameters in patients with high risk of diabetes type 2.	Monthly supplementation restored blood serum levels of 25(OH)D. However, this short-term treatment did not significantly improve the overall cardiometabolic parameters. The only significant findings were improvements in pulse wave velocity (PWV). PWV: ergocalciferol; −0.68 m/s (95% CI: −1.31, −0.05) and cholecalciferol; −0.73 m/s (95% CI: −1.42, −0.03).
Effects of vitamin D supplementation as an adjuvant therapy in coronary artery disease patients. [81] ChiCTR-TRC-13003958	Randomized Placebo-controlled Double-blinded *n* = 90	Coronary artery disease (SYNTAX score) Blood lipids BP HbA1c	To examine if daily supplementation with calcitriol (0.5 μg/day) for six months in stable CAD patients could enhance the SYNTAX score and cardiometabolic parameters.	Mean serum levels of 25(OH)D were restored in the intervention group. After six months, the interventional group had a significantly decreased SYNTAX score of −3.9 (95% CI: −6.6 to −0.7, *p* < 0.001). Furthermore, hsCRP decreased by −0.07 mg/dl (95% CI: −0.13 to −0.02, *p* < 0.001). However, the other secondary outcomes did not change significantly. The authors concluded that calcitriol supplementation is beneficial in CAD.
The effect of a single dose of vitamin D on glycemic status and C-reactive protein levels in type 2 diabetic patients with ischemic heart disease: a randomized clinical trial. [82] ACTRN12614000529640.	Randomized Placebo-controlled Double-blinded *n* = 95	hsCRP HbA1c Fasting blood sugar (FBS)	Whether a single dose of cholecalciferol (300,000 IU, i.m.) could improve glycemic status in type 2 diabetes patients.	Vitamin D status was restored after the intervention when compared to placebo. However, this study found no significant changes in vascular inflammation in the intervention group vs. placebo (hsCRP +1236.95 ng/mL (standard error 842.1, *p* = 0.3)). However, HbA1c was reduced by 0.48% (*p* = 0.04) in the vitamin D supplementation group. It was concluded that cholecalciferol improves glycemic status but fails to improve vascular inflammation.
A randomized controlled trial (RCT) evaluating the impact of targeted vitamin D supplementation on endothelial function in type 2 diabetes mellitus: The DIMENSION trial. [83] NCT01741181	Randomized Placebo-controlled Double-blinded *n* = 31	hsCRP Von Willebrand factor E-selectin Reactive hyperemia index	Whether daily supplementation for 16 weeks with cholecalciferol (either 2000 IU/day or 4000 IU/day) could improve vascular biomarkers and the reactive hyperemia index in patients with type 2 diabetes.	Vitamin D status was significantly improved in the intervention arm. After multivariate regression analysis, this study found no significant changes in parameters of endothelial function (*p* > 0.05).
The effects of vitamin D repletion on endothelial function and inflammation in patients with coronary artery disease. [84] NCT01570309	Randomized Placebo-controlled Double-blinded *n* = 90	hsCRP BPPro-inflamma-tory cytokines Adhesion molecules Endothelial function	To elucidate if a weekly supplementation with ergocalciferol (50,000 IU/week) for 12 weeks in patients with CAD could enhance endothelial function and vascular inflammation.	Mean average serum 25(OH)D was significantly raised in the intervention group vs. placebo. No significant improvements were demonstrated in markers of vascular inflammation (*p* = 0.79). Furthermore, blood pressure and all markers of endothelial function were not significantly impacted (*p* > 0.05). Hence, this study failed to find any cardiovascular benefits of weekly vitamin D supplementation.
Vitamin D inflammatory cytokines and coronary events: a comprehensive review [85].	Randomized Placebo-controlled Double-blinded *n* = 50	Adhesion molecules (VCAM-1, ICAM-1, E-selectin, VEGF) Pro-inflammatory cytokines (CRP, IL-6, IL-8, TNF-α)	To examine the cardiovascular effects of daily oral supplementation with cholecalciferol (4000 IU/day) for five days in patients presenting with acute MI and undergoing PCI.	Five days of cholecalciferol supplementation led to insignificant alterations in mean average serum 25(OH)D levels (*p* = 0.14). CRP underwent a significantly smaller increase in the intervention group vs. placebo (108.6% vs. 361%, *p* = 0.03). Likewise, a significant reduction in IL-6 (−31.6%; *p* = 0.05) was observed. VCAM-1 was reduced by 3.3% compared to a 23% increase in controls (*p* = 0.03). ICAM-1, E-selectin and VEGF were all insignificantly changed (*p* > 0.15). Thus, some inflammatory and adhesion markers were affected, while others were insignificantly impacted by vitamin D supplementation.
Effects of vitamin D supplementation on markers of vascular function after myocardial infarction—a randomized controlled trial. [86] EuDRACT ref: 2009-010367-17	Randomized Placebo-controlled Double-blinded *n* = 75	Blood pressure Cholesterol Reactive hyperemia index CRP Von Willebrand factor TNF-α E-selectinB-type natriuretic peptide	To investigate if two high-doses of orally administered cholecalciferol (100,000 IU) could improve cardiovascular markers in patients with a prior history of MI.	Serum 25(OH)D levels were only modestly improved after five days. No significant differences were observed in the reactive hyperemia index, systolic BP, diastolic BP or cholesterol levels. The only marker showing a significant change was CRP (−1.3 vs. +2.0 mg/L, *p* = 0.03). Thus, high-dose vitamin D supplementation insignificantly improved cardiovascular markers after MI.
Calcium/vitamin D supplementation and coronary artery calcification in the Women’s Health Initiative. [87] NCT00000611	Randomized Placebo-controlled Double-blinded *n* = 754 All women	Coronary artery calcium (CAC) score	To assess the vascular effect of daily supplementation with calcium (1000 mg/day) + cholecalciferol (400 IU/day) for an average of seven years in women. The objective was to evaluate if supplementation could alter the plaque burden in the intervention group.	Serum vitamin D status at baseline and after intervention was not obtained in this study. The study found no significant changes in CAC score. Adjusting the multivariate odds ratio (OR) for incremental CAC scores failed to demonstrate a significant reduction in plaque burden (*p* > 0.05).
Long-term vitamin D supplementation affects metabolic status in vitamin D-deficient type 2 diabetic patients with coronary artery disease. [88] IRCT201510315623N56	Randomized Placebo-controlled Double-blinded *n* = 60	Fasting blood glucose hsCRP Plasma NO Serum insulin	Sought to examine if supplementation with 50,000 IU cholecalciferol every second week for six months could improve vascular inflammation and glycemic markers. Participants were type 2 diabetic patients with CAD.	Vitamin D status was markedly improved in the intervention arm during follow-up. The results showed a significant attenuation in vascular inflammation (including hsCRP and plasma NO reductions, *p* < 0.05) and improved glycemic status in diabetic patients supplemented with vitamin D for six months.
The effects of vitamin D and probiotic co-supplementation on mental health parameters and metabolic status in type 2 diabetic patients with coronary heart disease: a randomized, double-blind, placebo- controlled trial. [89] IRCT2017073033941N4	Randomized Placebo-controlled Double-blinded *n* = 60	Mental health parameters hsCRP Plasma NO Glycemic control HDL cholesterol	To investigate if combined vitamin D (50,000 IU) + probiotic supplementation every second week for 12 weeks could be beneficial regarding cardiovascular parameters in type 2 diabetic patients with CAD.	Combined vitamin D and probiotic supplementation restored serum 25(OH)D status (*p* < 0.05). Vitamin D + probiotics seemed to significantly improve vascular inflammation (lower hsCRP) and glycemic markers.
